# The Mode of Securing Medical Education

**Published:** 1879-07

**Authors:** 


### Editorial.

### THE MODE OF SECURING MEDICAL EDUCATION.

The profession of medicine has one great drawback in the board of medical impostors which infest it. It is not there altogether from a want of thoroughness in the education of those who make medicine a regular study, but from a want of the means of protecting the profession and the public against those who never pretended to study at all. The avenues through which interlopers enter the profession and legalize their position are numerous. Diplomas are readily obtained from regularly chartered institutions without any medical knowledge at all ; and although such colleges, so-called, may not be recognized by honorable institutions or deserving medical men, yet they show the evidences of authority, and the legal right to offer their professional services to the public is the same as the most thoroughly educated physician. Hundreds of these bogus diplomas have been sent out from Philadelphia without the recipient ever having been seen by the operators of the concern.

"The University of Philadelphia" is a name calculated to deceive the unwary into the idea of the parchment having been issued from that old reliable Institution, The University of Pennsylvania. Thus for a few dollars, a man without any knowledge of medicine imposes himself on a community and brings reproach on a profession to which he does not belong. We would not be understood as denying that many colleges who claim not only the legal right to confer the degree, but respectability among medical colleges, virtually sell the authority to practice medicine. We have good reason for believing this is the case and that it will continue so long as our present system of examinations prevails.

The system of State licensing, as now pursued in Georgia, is not less outrageous than the Philadelphia diploma selling. Students with very poor preliminary study and one or two months in college, have been known to return home with as perfect legal authority to practice as any man in the profession. Now while we object to the time of study having anything to do with admission to examination, this is mentioned to show the impossibility of preparation in so short a time, and that improper persons are introduced through this system of State licensing by a board feeling no particular responsibility or interest in the matter other than their fee for the certificate.

Again we have one ideal systems of medicine, which furnish quite a number of practitioners not thoroughly versed in all branches of medical science. If the homœopath and eclectic can impress their peculiar doctrine, it is believed to be sufficient to make a practitioner, and hence such are sent out upon the world with very little real medical preparation.

To cure all these evils, or rather to stop these fraudulent inlets to a noble and useful profession, by which it is brought into disrepute, we earnestly insist upon a thorough change in the mode of admission. Let us stop the quarrel about time of study, the length and number of college courses and the season of year for teaching. These cannot insure preparation nor avert the trouble of interlopers. Let us have a medical board in every State to examine all who apply for admission into the profession, whether fresh from the college or whether he has been to college at all. Let his thorough knowledge of all the branches of medical science be tested by this board. The itenerant quack, the eclectic and homœopath and all alike, must come to the test or be debarred by State law from the practice of medicine. This will work no hardship to any who are qualified for professional duties, but serve as a protection to them and the community.

In order to be effective, the State Board should be appointed by the Governor, approved by the Senate, paid by the State, and subject to removal for failing to perform properly their prescribed duties, which, like other State officers, they should be sworn to do. That any failure to hold applicants to rigid account, or injustice shown in refusing any, may be detected, the examinations should be conducted in writing, and each filed for inspection.

The professional State tax now imposed upon physicians in Georgia will amply pay a board to discharge this duty, and also to supervise and direct the sanitation of the State. If men are appointed to serve the public, they should be paid. The honor of certain non-paying offices may be sought, but duties efficiently performed need not be expected without compensation, and the power to accomplish what is undertaken. Evidence of this is found in the failure of the State Board of Health to perform the duties assigned it. The appointees accepted and continue to wear the honor conferred, but in the matters of vital statistics and general sanitation, the board has proven an utter failure. The difficulty did not exist in a want of professional ability, nor disposition on the part of members to discharge their duties. The registration of births and deaths in order to afford reliable, vital statistics must be accurate and universal. Without the aid of others, of course, the board cannot obtain these records, and the law under which they acted, did not secure such assistance. With this additional duty made incumbent on tax receivers, it is believed that vital statistics may be accurately obtained. In regard to sanitation the board was not more successful, nor need anything in that way be expected without compensation for the board and means to pay sanitary expenses.

The medical examining board of the State, above proposed, to consist of salaried officers, can very well act as supervisors of sanitation and vital statistics, so that every public duty connected with State medicine may be concentrated in one salaried body, held strictly accountable to the State for their acts.

We now have various irresponsible bodies to perform these duties, and the result is that the public and profession of medicine are very little benefitted by them.

With three or four college faculties annually granting diplomas, and two or three examining boards granting licenses to practice medicine at a fee of twenty dollars, each member of which, perhaps, granting all through the year, temporary license at a fee of five dollars, and the nine members of the State Board of Health, we have about forty men poorly paid and paid not at all, to imperfectly do the work which may be effectually done by nine. By all means give us an effective State Medical Board.

